# Supplementary material for: Are carbon emissions trading and green financial instruments synergistic? -Comprehensive quantitative research based on content analysis
Source: PLoS One. 2024 Mar 7;19(3):e0298601. doi: 10.1371/journal.pone.0298601 (PMC10919601; doi:10.1371/journal.pone.0298601)
Supplement: S1 Data — (ZIP) [file pone.0298601.s001.zip › Data/地级市Z_F工作报告.zip!/╣π╢1⁄2/╔╟╬▓╩╨/└┤╫╘╣1⁄2╓┌║┼í╛┬φ┐╦╩2╛▌═°í┐_2016.docx]

各位代表：

我代表市人民检察院向大会报告工作，请予审议，并请列席会议的同志提出意见。

2015年，全市检察机关在市委和省检察院正确领导下，在市人大及其常委会监督支持下，深入贯彻落实党的十八大和十八届三中、四中、五中全会及习近平总书记系列重要讲话精神，忠实履行宪法和法律赋予的职责，深入推进检察改革和队伍建设，为我市改革发展稳定提供了有力司法保障。

一、着力服务大局，促进经济社会平稳较快发展

充分发挥打击、预防、监督、教育、保护职能，助推良好的营商、政务、社会环境建设。

——专项预防、同步预防与查办案件相结合，排查化解矛盾，推进工业园区与重点工程建设。认真开展“潮惠高速公路汕尾段建设工程”专项预防，对存在问题较多的路段，提出依法解决征地拆迁、阻工问题的建议，力促工程推进。对重点工程、产业园区建设中发生的群访闹访介入调查，依法查处侵吞征地拆迁补偿款侵害农民利益的案件，共立案查处此类职务犯罪案件6件10人,如查办了红草镇水陂村原村委主任黄隆冠挪用征地补偿款案。

——引建良好的渔业管理秩序，服务我市海洋经济“一带一路”建设。集中力量查办了一批海洋渔业职能部门工作人员与中介相互勾结，在办理渔民上岗证、船长证、体检、培训等环节利用职务之便收受贿赂的窝案串案，深挖国家惠渔柴油补贴申领、审批环节存在的腐败问题，立案查处26件26人。

——督促纠正资源环境领域违法犯罪行为，保护绿色生态。如对陆河县乱砍滥伐林木涉嫌犯罪被放任一线索进行立案监督，督促林业公安立案并追缉逃犯。对汕尾电视台“有话直讲”栏目曝光的品清湖环境污染问题进行跟进，督促相关职能部门依法履职。

——注重从行政执法监督中开拓案源，纠正不作为、乱作为，推进依法行政、优化政务环境。如在政府采购环节，对政府采购主体、采购职能部门的违法行为提出书面检察建议；对行政部门怠于履职，造成国有划拨土地使用权被非法转让、受让人违法建设等问题进行监督，运用柔性手段纠正行政违法。

——积极参与“禁毒暨打击整治涉枪犯罪”专项行动，推进治安重点问题治理。加大对毒品案件的证据把关力度，实行毒品案件批捕阶段全面提审、提审过程全程同步录音录像，起诉环节自行补充侦查等制度。牵头公安、法院等单位召开毒品犯罪案件办理工作座谈会，对存在的问题达成解决共识，形成打击合力。共批捕毒品犯罪嫌疑人790人，起诉被告人856人，起诉毒品犯罪人数同比上升12 %；批捕涉枪犯罪嫌疑人29人，起诉被告人22人。如批捕起诉了刘汉周等人贩卖、制造毒品案，该案缴获冰毒25公斤、液态冰毒1200多公斤，枪支7支，子弹128发。

二、着力维护社会和谐稳定，促进平安汕尾建设

坚持以人民群众平安需求为导向，依法打击严重刑事犯罪，维护国家安全和社会稳定。

——突出打击影响社会稳定的刑事犯罪。根据我市社会治安状况，严厉打击危害国家安全、公共安全犯罪和涉恐犯罪，从严打击严重暴力、多发性侵财及黑恶势力犯罪。对其中的重特大、社会影响大的案件提前介入，引导侦查取证50件80人，从快从严惩处。全年批捕各类刑事犯罪嫌疑人1916人、起诉被告人2115人。如批捕起诉了支建故意杀人案，陈楚霞入室盗窃案。

——依法做好矛盾化解和息诉工作。贯彻宽严相济刑事政策，对轻微犯罪轻缓化处理，不批捕22人，不起诉108人。做好矛盾纠纷的化解和调处工作，对犯罪嫌疑人认罪悔罪、积极赔偿的轻微刑事案件，促成和解29件36人。加大对错误的检察处理决定和生效刑事裁判监督纠正力度，复查4件4人，纠正2件2人。加强信访接待室规范化建设，采取约访下访、远程视频接访等方式，着力解决控告难、申诉难问题。全年共办理群众信访306件次，其中检察长接待来访群众20批51人次。

——积极参与社会治安防控体系建设。发挥派驻检察室和检务工作联络室贴近群众的特殊作用，开展违法调查、化解矛盾、监督基层执法，提出防范建议。依法惩治利用网络实施的诽谤、敲诈勒索、淫秽色情和侵害公民个人信息等犯罪，净化网络环境。创新未成年人检察工作，落实专人办理、心理干预、案后帮教等措施，为罪错未成年人回归社会提供帮助。广泛开展检察官进机关、进乡村、进社区、进学校、进企业，促进公民自觉守法。

三、着力惩治预防职务犯罪，促进反腐倡廉建设

坚决贯彻中央、省、市委关于反腐倡廉的决策部署，集中力量惩治职务犯罪，提高反腐败法治化水平。

——加大查办案件力度。坚持有腐必反、有案必办，全年共立案侦查职务犯罪案件71件75人，同比分别上升29%和10%。其中贪污贿赂案件49件53人，渎职侵权案件22件22人。围绕“查办和预防涉农领域职务犯罪”专项工作，重点查办涉农领域职务犯罪，共立案53件57人；继续深挖涉毒“保护伞”职务犯罪，立案2件2人。全年查办大案要案21件21人，其中科级以上16人，正处级2人。如立案侦查了原城区政协主席刘妈金贪污案；根据省检察院指定管辖，立案侦查了原省水利厅东江分局局长（原惠州消防局局长）杨锦财行贿、巨额财产来源不明案。深入开展追逃追赃工作，共追逃、劝投涉案在逃嫌疑人5人。查办职务犯罪案件数量在全省排名有所提前，查办大要案有了新的突破，形成了惩治职务犯罪高压态势。

——提高精准办案能力。制定《不立案举报线索审查评估工作规定》，加强对案件线索的管理和评估，提高举报线索的利用价值和成案率。市检察院发挥侦查一体化机制优势，统筹调动全市办案力量，以交办、参办、提办和督办方式，攻坚突破重大疑难复杂案件和跨地区案件，带头查办大案要案。加强人权司法保障，严格规范行使侦查权，依法适用强制措施，依法扣押冻结涉案款物，严格执行讯问犯罪嫌疑人全程同步录音录像制度，适应“镜头下”办案。

——深化职务犯罪预防。发挥3个职务犯罪警示教育基地作用，举办反腐倡廉专题讲座52场，受教育人员4500人次。对全市198个重点建设项目开展专项预防，审查重点工程建设项目招标文件197件。书面提出加强监管、完善制度的检察建议3件，口头提出预防建议195次。深化商业贿赂源头治理，提供行贿犯罪档案查询747次。运用预防职务犯罪年度报告，揭示犯罪特点和规律，剖析原因，提出建议。

四、着力发挥法律监督作用，促进法治汕尾建设

坚持严格依法办案，防止冤错案件，努力让人民群众在每一个司法案件中都感受到公平正义。

——切实强化刑事诉讼监督。正确把握捕诉标准，努力做到不枉不纵、不错不漏。对不构成犯罪的，事实不清、证据不足的，不批捕131人，不起诉29人；监督侦查机关立案4件、撤案26件，纠正漏捕5人、漏诉3人，追加漏罪18人；对滥用强制措施和违法取证，提出纠正意见16件次。推进“两法衔接”平台的启动与应用，实现省、市、县三级联网共享，监督行政执法机关移送犯罪线索3件。对刑事裁判提出抗诉7件12人，同比上升16.7%。

——开展有特色的民商事、行政法律监督。对引发上访、群访未进入诉讼的民事、行政案件，通过调查发现违法行为，依法纠正以化解矛盾。深入挖掘民商事案件背后的行政违法行为，向相关部门提出检察建议6件。对一涉及国有资产流失3000多万元的案件，已上报省检察院备案准备提起公益诉讼。

——强化刑事执行监督，维护监管秩序。核查羁押期限案件2121件，提出监督意见34次；开展死刑执行临场监督3批12人。加强羁押场所秩序的整顿，配合省检察院监所处对海丰县看守所、海丰县检察院驻所检察室开展巡视检察，对存在的突出问题进行专项整治。加强社区矫正法律监督，依法提出纠正意见。重视“特赦”工作，审查并出具同意“特赦”检察意见42份。

——全面加强自身监督。配合市人大常委会对刑事诉讼监督工作进行专项视察并报告工作。举办检察开放日活动，邀请人大代表、政协委员视察，邀请媒体采访、群众参观，主动接受人大监督、民主监督和舆论监督。注重发挥人民监督员、规范司法行为监督员监督办案的重要作用。完善案件集中管理机制，全面运用全国统一业务应用系统，对办案活动实行网上全程、统一、实时、动态监管。

——深入推进检察改革。围绕以司法责任制为核心的四项重点改革任务，成立领导小组和3个专题调研组，开展调研论证，并通过会议、专家授课准确解读司法改革相关政策，为推进改革做好思想和工作准备，确保改革过程中工作不乱、人心不散。扎实推进涉法涉诉信访工作机制改革，坚持诉访分离，落实依法终结制度。深化检务公开改革，推进职能职责公开向办案信息公开转变。开展举报宣传周活动，加强检察门户网站建设，开通官方微博微信（正义汕尾），以全国检察机关案件信息公开系统为平台，已公开案件信息2659件和终结性法律文书837篇。

五、着力落实从严治检要求，促进过硬队伍建设

始终把队伍建设摆在各项工作的首位，坚持从严教育、从严管理、从严监督。

——扎实推进教育实践和专项整治活动。严肃认真开展“三严三实”教育实践活动和规范司法行为专项整治工作，突出“严实”要求，整改执法司法突出问题。领导班子成员分专题为干警作辅导课，发挥带学促学作用。市检察院在搞好自身教育整改的同时，注重加强对县（区）院的指导，检察机关的工作作风和检察队伍的精神面貌有了明显改进。

——全面加强素质能力建设。组织全市检察干警共251人次前往高校进行各类培训；切实落实检委会集体学习制度，确定学习专题，采用检委会委员和业务骨干讲解、邀请省院业务专家讲授课程等多种形式组织集体学习；举办第四届汕尾市优秀公诉人竞赛，与汕尾法学会合作办好《汕尾法学》检察专刊。通过上述举措，队伍的整体素质和业务能力有了明显提高。

——抓常抓细纪律作风建设。认真贯彻市委和上级检察院的部署，逐级签订责任状和保证书，把党风廉政建设的主体责任、监督责任落到实处。采取下级院检察长向市检察院汇报工作等方式，加大对下级院领导班子特别是“一把手”的监督力度。持续开展常态化检务督察，确保严格执行中央八项规定和检察纪律禁令。认真开展“全面深化检务整治、正风肃纪集中行动”专项工作，加大正风肃纪力度。全年刑事立案查处2名检察人员，政纪立案调查3名检察人员，并及时通报典型案例，扩大警示教育效果。

另外，我们还认真落实《全国基层检察院建设规划》，用好专项资金，四县（区）院基础设施水平有较大的提升。市院办案和侦查技术用房建成并投入使用，完成法警训练场地建设；购置信息数据恢复设备和侦查用车等技术装备，完成机房建设和涉密信息分级保护工作，办案条件和科技水平得到显著改善。同时，积极整合两级院法警力量，为办案安全提供强有力的保障和支持。

检察工作在过去一年中所取得的成绩，是市委坚强领导、市人大及其常委会依法监督的结果，是各级政府、政协、社会各界和人大代表关心、支持、帮助的结果。在此，我代表市人民检察院表示衷心的感谢！

面对新形势和新期待，我们清醒地认识到，我市检察工作还存在一些问题和不足。一是检察职能作用的发挥与服务大局的要求还有一定差距；二是法律监督工作中的薄弱环节需要努力补强；三是司法规范化水平有待进一步提升；四是检察体制改革措施尚未全面落实到位，配套方案还要进一步细化完善；五是案多人少的矛盾日益突出，队伍管理工作仍需继续改进。对此，我们将坚持问题导向，采取有力措施加以解决。

新的一年，全市检察机关将继续强化法律监督、自身监督和队伍建设，坚持以法治为引领、以办案为中心、以改革为动力，不断提升司法能力和工作水平，努力创造安全稳定的社会环境、公平正义的法治环境、优质高效的服务环境。

一要进一步深化服务大局举措。坚持把检察工作摆到经济社会发展全局中谋划，切实服务“五大发展理念”在汕尾的落地，深入研究市委“三大抓手”、“三大民生”、“三大环境”战略推进过程中叠加的相关法律问题，增强工作前瞻性、主动性，准确把握检察工作切入点和结合点，更加有效服务保障我市“十三五”规划顺利实施。

二要进一步参与平安汕尾建设。充分履行批捕、起诉职能，依法严厉打击危害国家安全、公共安全的犯罪，加大对危害食品药品安全、危害生产安全、涉枪、涉毒等犯罪惩治力度，深化立体化社会治安防控体系建设。

三要进一步突出法律监督职能。贯彻对权利的司法保障、对权力的司法监督要求，继续强化对侦查活动的监督，促进依法办案、保障人权；全面加强刑事执行监督，依法保障被刑事执行人合法权益；推进行政检察工作，探索实施检察机关提起公益诉讼制度；健全行政执法和刑事司法衔接机制，加强对行政执法活动的监督。

四要进一步加大惩治职务犯罪力度。敢于担当、主动作为，继续减少职务犯罪存量，控制增量。重点查办市场监管、社会管理、公共服务提供、扶贫开发岗位的贪污、贿赂、失职、渎职犯罪，查处发生在群众身边、侵害群众利益的职务犯罪，查处和预防县乡换届选举中的职务犯罪。完成职务犯罪预防教育基地建设，扩大警示面。

五要进一步深入推进检察改革。顶层设计与汕尾实际相结合，统筹安排，确保五月份以司法责任制为核心的“四项改革”实施运行。继续深化刑事案件速裁程序、涉法涉诉信访、检务公开等改革。

六要进一步强化过硬队伍建设。全面贯彻从严治党、从严治检要求，以正规化、专业化、职业化为方向，努力建设一支忠于党、忠于国家、忠于人民、忠于法律的检察队伍。创新教育培训工作，加大招录、引进人才力度，提高队伍司法能力和专业化水平。落实好主体责任、监督责任，持续整治“四风”和司法作风突出问题，树立检察人员公正廉洁的良好形象。

各位代表，新的一年，全市检察机关决心在市委和省检察院的坚强领导下，认真落实本次会议的决议要求，创新进取，真抓实干，忠实履行好维护社会大局稳定、促进社会公平正义、保障人民安居乐业的职责使命，为建设平安汕尾、法治汕尾、美丽汕尾作出新的更大贡献！
